# Changes in Cabernet Sauvignon yield and berry quality as affected by variability in weather conditions in the last two decades in Lebanon

**DOI:** 10.1038/s41598-024-57665-z

**Published:** 2024-03-24

**Authors:** G. Ghantous, K. Popov, Z. El Sebaaly, Y. N. Sassine

**Affiliations:** 1 Department of Agronomy, Faculty of Agronomy, University of Forestry, Sofia, Bulgaria; 2 Department of Plant Production, Faculty of Agronomy, Lebanese University, Beirut, Lebanon

**Keywords:** Climate change, Winegrapes, Production, Wine quality, Regression analysis, Physiology, Plant sciences

## Abstract

Cabernet Sauvignon is the most planted cultivar in Lebanese vineyards. This study investigated the variation of its production at two vineyards ‘Kanafar’ (West Bekaa at 1020 m.a.s.l) and ‘Taanayel’ (Central Bekaa at 800 m.a.s.l) and their interactions with weather conditions from 2006 till 2018. Evaluation of climate records denoted interannual variability in weather conditions occurring in 2015 in Kanafar and in 2008 in Taanayel. Average yield peaked in 2009 in Kanafar (19,187.0 kg ha^−1^) and in 2011 in Taanayel (14,279.0 kg ha^−1^), both years marked a turning point after which values of average yield shifted downwards (by 31–67% in Kanafar, and 14–82% in Taanayel). At Kanafar, after 2015, averages of yield, weight of 200 berries (W200B), potential alcohol (PA), and total polyphenolic richness (TPR) decreased by 35%, 1.5%, 36.2 g, and 50%, respectively. In Taanayel, only TPR content in berries was significantly affected by varying weather conditions (decrease by 20%). Also, TPR values followed a progressive decreasing pattern starting from 2006 at both vineyards with minor exceptions. Multiple regression analysis assessed the relationship between various indicators and weather variables at each vineyard. It showed that the decrease in yield at Kanafar correlated with higher temperature during the growing season (by 0.6 °C), higher solar radiation from early-spring to early-summer (by 13.9–27.1 W m^−2^), and lower values of maximum wind speed during mid to late summer (by 0.4 m s^−1^), occurring during 2016, 2017, and 2018 at Kanafar. The model explained 60% of yield variations at this vineyard. Further, weather variables accounted for 61% (*R*^2^ = 0.61) of changes in PA and for 58% (*R*^2^ = 0.58) of TPR of berries at Kanafar. Conclusively, interannual variability in weather conditions had more serious negative influence on Cabernet Sauvignon production at Kanafar than at Taanayel, but had a similar negative influence on polyphenols accumulation in berries, and thus on potential wine quality produced at both vineyards.

## Introduction

As a Mediterranean country, Lebanon is characterized by its fine wines The remarkable history of wine making activity in Lebanese wine began in the Phoenician era. Today the number of wineries in Lebanon has reached sixty with a total of eight million bottles produced per year^1^. The latest survey conducted on the state of vitiviniculture in Lebanon reported that the cultivar Cabernet Sauvignon, originating in France, is the most planted variety and vineyards cultivated by winegrapes are mostly concentrated in the Bekaa Valley (56% of vineyards)^2^. Lebanon is characterised by a Mediterranean climate: hot, dry summers with low precipitation levels from June to September and cool, rainy winters from December to mid-March with an average annual temperature of 15 °C^3–5^. Along the coast, summers are hot and humid, with temperatures crossing 35 °C in August. About 70% of the average rainfall in the country falls between November and March. January is the coldest month, with temperatures around 5 to 10 °C, and it records the maximum amounts of rainfall^6^. On the coast, the mean annual rainfall is between 700 and 1000 mm. With snow in the mountains, inland Lebanon experiences more precipitation (1600 mm) than the coast^4^.

Climate change has been posing problems to the viticulture sector worldwide. Changes in climate conditions in winegrapes producing regions led to producing longer growing seasons and shorter dormant periods^7^. These changes however, are not constant across regions and seasonal cycles^8^. According to Bateman^9^, 2010–2019 has been the hottest recorded decade of the twentieth century, and the IPCC^10^ predicted an increase in drought frequency and intensity in the Mediterranean region in the twenty-first century, which is projected to be due to a decrease in precipitation coupled with an increase in temperature in this region. In Lebanon, drought periods are expected to be 9 days longer by 2040, and 18 days longer by 2090^11^.

Climate factors like solar radiation, rainfall, and temperature have a major influence on the grapevine physiology, growth, phenological development, yield and grape berry quality^7,12–14^. Therefore climate has a significant influence on grape ripening and on final characteristics of wines^15^. Winegrape cultivars have different responses to varying climate conditions because they have different demands for sunlight, heat, and water^8,16,17^. Selection of winegrape cultivars by winegrowers has classically relied on the cultivars’ phenotypic traits, capacity to produce consistent yields, and capacity to reach an appropriate balance of sugar, acid, and other compounds under local climatic conditions^18^.

The objective of the current study was to conduct an integrative analysis of climate, production and berry quality of Cabernet sauvignon, a representative variety widely-grown in West-Bekaa (Kanafar vineyards) and Central Bekaa (Taanayel vineyards), throughout thirteen consecutive years (2006 until 2018). The aim was to evaluate whether there was a change in prevailing climate conditions at the selected vineyards and whether such variation has had an impact on the performance of the cultivar’s growth, production, and quality.

## Materials and methods

### Vineyards and timeframe

This study was conducted in the vineyards of ‘Chateau Ksara’ situated in West Bekaa, Kanafar at an elevation of 1020 m.a.s.l, longitude: 35° 43′ 1E, and latitude 33° 38′ 26N on a surface of 8300 m^2^, and Central Bekaa, Taanayel, located at an elevation of 800 m.a.s.l, longitude: 35° 52′ 16E, and latitude 33° 48′ 2N on a surface of 3140 m^2^. Those two areas were planted with the cv Cabernet Sauvignon at 2.5 m × 1.25 m spacing. The rootstock was 110 Richter (R110). Vines were initially trained with double Guyot technique and pruned leaving twenty buds per vine.

### Climate data

Weather variables assessed included temperature, relative humidity (RH), precipitation, solar radiation (SR), average wind speed (AWS), and maximum wind speed (MWS). Climate data used included annual means of climate factors as well as means during different intervals of the production cycle of Cabernet Sauvignon; growing season (March–September), early-mid spring (March–April), late-spring to early-summer (May–June), mid-summer to late-summer (July–August), mid-summer to early-autumn (July–August–September), and late-summer to early-autumn (August–September) for a comprehensive evaluation of the effects of these seasonal climate factors on Cabernet Sauvignon tested indicators. Climate data for 2006 till 2018 was sourced from meteorological stations (Kanafar, Haouch Ammiq, and Tal-Amara stations) of the Lebanese Agricultural Research Institute (LARI). It was provided as daily values, then monthly and annual means were calculated relatively to each year of study.

### Data collection

One hundred random vines were selected from each region as a sample to record the effect of climate factors on quantitative and quality variables of Cabernet Sauvignon. Data was recorded by the engineers of Chateau Ksara from 2006 till 2014. Researchers from the Lebanese University continued the data collection in collaboration with Ksara winery and following methods adopted there from 2015 till 2018.

Harvested yield was recorded per plant and then expressed as kg ha^−1^. Harvest dates were determined based on fruit chemistry analysis after measuring the potential alcohol (PA) by volume (%v/v) using a digital refractometer (PR101, Atago, Bellevue, WA, USA) and according to the ITV database. Grapes were harvested when potential alcohol was between 12 and 14%.

A random sampling of 200 berries was done for multiple times (a minimum of three times), starting from the beginning of the veraison stage until reaching a PA percentage falling in the range of 12–14%. At full berry ripeness, the weight of 200 berries (W200B) was recorded in grams. Titratable acidity (TA) was measured using the acid/base titration, using NaOH 0.1 N and bromothymol blue (4 g L^−1^) as an indicator dye.

For further measurements, samples of 500 g of fully ripened berries were used to evaluate the anthocyanins and phenolic compounds after harvest, following the ITV (Institut Technique de la Vigne et du Vin) method^19^.

The concentration of anthocyanins (Ant) and the total anthocyanin potential (TAP) were estimated as follows:

$${\text{Anthocyanins }}\left( {{\text{mg L}}^{{ - {1}}} } \right) \, = {\text{ OD}}_{{{520}}} \, \times { 22}.{75 } \times { 2}0$$

$$\begin{aligned} {\text{Total anthocyanins potential }}\left( {{\text{mg kg}}^{{ - {1}}} } \right) & = {\text{ Anthocyanins }}\left( {{\text{mg}}\;{\text{L}}^{{ - {1}}} } \right) \, \times { 1}00 \hfill \\ & \quad \times \, \left( {{\text{weight of grape juice }}\left( {{\text{mg}}} \right) \, + { 1}00} \right)/\left( {{\text{weight of grape juice }}\left( {{\text{mg}}} \right)} \right). \hfill \\ \end{aligned}$$

Glories method was applied for estimating anthocyanins and total phenolic contents as follows: An aqueous solution pH 3.2 was prepared by adding 5 g of tartaric acid to water (1 L), and the pH was adjusted to 3.2 by NaOH. Then, 15 mL of this solution were added to a first sample of 50 g of grape juice. Also, a solution pH 1 was prepared by adding HCL (37%) in distilled water and adjusting the pH to 1, and 15 mL of this solution were added to a second 50 g sample. Following a four-hour maceration at 20 °C, the samples were filtered through glass wool. Based on this method, measurement of anthocyanin is based on anthocyanin discoloration by SO_2_. Therefore, 1 mL of each prepared filtrate (pH 1 and pH 3.2) was added to 1 mL of ethanol (0.1%) and 20 mL of concentrated HCl (2%). Then, 10 mL of the mixture were mixed with 4 mL of distilled water and put in the first tube. Besides, the other 10 mL of the mixture were mixed with 4 mL of sodium bisulfite (15%) and put in a second tube. Twenty minutes later, the optical density was measured for both tubes at 520 nm against distilled water.

Anthocyanin concentration (Ant) was determined in mg L^−1^ as follows:

$${\text{Ant }}\left( {{\text{mg L}}^{{ - {1}}} } \right) \, = { 875 } \times \, \left( {{\text{OD tube 1 in water}} - {\text{OD tube 2 in bisulphite}}} \right)$$

(875 being the slope of the calibration curve obtained from malvidin-3-glucoside).

Following this calculation, two values were calculated as Ant1 and Ant2. Then, the potential of easily extractable anthocyanins (PAE) and Anthocyanin Extractability (AE) were calculated according to Ribéreau-Gayon et al.^20^, as follows:

$${\text{AntpH3}}.{2 } = {\text{ Dilution factor }} \times {\text{ Ant2}}$$

$${\text{AntpH1 }} = {\text{ Dilution factor }} \times {\text{ Ant1}}$$

$${\text{PAE }} = \, \left( {{\text{AntpH3}}.{\text{2/AntpH1}}} \right) \, \times { 1}00$$

$${\text{AE }} = \, \left[ {\left( {{\text{AntpH1 }} - {\text{ AntpH3}}.{2}} \right){\text{/ApH 1}}} \right] \, \times {1}00$$

To estimate total phenolic richness (TPR) in the extracts macerated at pH 3.2, a dilution to 1/100 was performed and the optical density was measured at 280 nm against distilled water. Then total phenolic richness was calculated:

$${\text{TPR }} = { 2 } \times {\text{ OD}}_{{{28}0}} \times { 1}00$$

### Statistical analysis

Different statistical tests were applied using the SPSS program version 26 to analyze the data at a 95% confidence level.

Cluster analysis was done applying a divisive hierarchical algorithm and average group method using the squared Euclidean distance for dividing years (2006 till 2018) into separate clusters. The variables included in the analysis were temperature, precipitation, SR, RH, AWS, and MWS (mean annual values and means during the different intervals of the production cycle) to check for possible variability in weather conditions at each vineyard during the study timeframe. Each cluster included a separate set of years showing more or less comparable means of the predetermined climatic factors. The contribution of each climatic factor in the cluster analysis was determined using the factor analysis option provided by the SPSS program.

Also, an *ANOVA* test was performed to investigate the separate and combined effects of the factors ‘vineyard’ and ‘year’ on the indicators studied. Furthermore, a *t-test* was performed for comparing means of the Cabernet Sauvignon indicators, and of climate predictors (found as main contributors by the factor analysis) between the distinct groups of years identified by the cluster analysis at each vineyard. Duncan Multiple Range test was applied for mean comparisons of Cabernet Sauvignon indicators during the whole period of study (2006–2018).

### Regression analysis

A multivariate regression model was used to confirm the impact of the ten influential climate predictors which were found as exhibiting a great influence on the grouping of the thirteen years into separate clusters at both vineyards (Kanafar and Taanayel). These climate predictors were used to estimate the quantitative relationships between grape indicators and climate at each of the vineyards studied. Models were developed only for indicators that showed a statistical difference (at *P*_*value*_ < 0.05) in terms of mean values between the two groups of years delineated by the cluster analysis at each vineyard.

Linear relationships between grape yield, AW200B, TSS, TA, TAP, EA, and TPR and climate predictors were developed to determine the changes in these indicators due to changes in predetermined climate predictors during the study period (2006 to 2018).

These relationships were derived as follows:

$$\begin{aligned} \Delta {\text{Y}} & = {\text{ constant }} + \, \left( {\upbeta {1 } \times \, \Delta {\text{CF1}}} \right) \, + \, \left( {\upbeta {2 } \times \, \Delta {\text{CF2}}} \right) \, + \, \left( {\upbeta {3 } \times \, \Delta {\text{CF3}}} \right) \, \hfill \\ & \quad + \, \left( {\upbeta {4 } \times \, \Delta {\text{CF4}}} \right) \, + \, \left( {\upbeta {5 } \times \, \Delta {\text{CF5}}} \right) \, + \, \left( {\upbeta {6 } \times \, \Delta {\text{CF6}}} \right) \hfill \\ & \quad + \, \left( {\upbeta {7 } \times \, \Delta {\text{CF7}}} \right) \, + \, \left( {\upbeta {8 } \times \, \Delta {\text{CF8}}} \right) \, + \, \left( {\upbeta {9 } \times \, \Delta {\text{CF9}}} \right) \, + \, \left( {\upbeta {1}0 \, \times \, \Delta {\text{CF1}}0} \right) \hfill \\ \end{aligned}$$

where, ∆Y is the observed change in grape indicators (yield/W200B/TSS/TPR) and β 1–10 are the coefficients of weather variables respectively, and ∆CF1-10 are the observed changes in weather variables, respectively, during 2006 till 2018.

## Results and discussion

### Effect of vineyard and years

Results of the *ANOVA* test (Table 1) showed that the separate effect of the factor ’vineyard’ was significant (*P*_*value*_ < 0.05) on most indicators, except W200B, TSS, and PAE, and the separate effect of years was significant on the majority of indicators, except on TA. The combined effect of both factors vineyard*year was significant on most indicators, except TSS and TA.

**Table 1:** Separate and combined effects of vineyard and year on Cabernet Sauvignon production and quality (*P*_*value*_ < 0.05).

	*P*_*value*_ Yield	*P*_*value*_ W200B	*P*_*value*_* TSS*	*P*_*value*_ TA	*P*_*value*_ TAP	*P*_*value*_ TPR	*P*_*value*_ AE
Vineyard	0.00	0.47	0.54	0.00	0.00	0.01	0.92
Years	0.00	0.00	0.00	0.19	0.00	0.00	0.00
Vineyard*year	0.00	0.00	0.10	0.72	0.00	0.00	0.00

W200b: weight of 200 berries; TSS: total soluble solids; TA: titratable acidity; TAP: total anthocyanin potential; TPR: total polyphenolic richness; EA: extractible anthocyanins.

### Cluster analysis

Cluster analysis based on weather variables (Table 2) separated years into two groups at each vineyard. Years grouped in a same cluster are more similar to each other than to those in the other cluster. Therefore, years grouped in cluster 1 had more similar weather conditions to each other than to those of cluster 2. In Kanafar, cluster 1 enclosed the years from 2006 till 2015, and cluster 2 comprised the years from 2016 till 2018. In Taanayel, cluster 1 grouped the years 2006, 2007, 2008 and 2013, whilst cluster 2 grouped the years 2009 till 2018 (excluding 2013).

**Table 2:** Clusters of years obtained at the vineyards of Kanafar and Taanayel and observations included in each of them based on the average method.

Kanafar			Taanayel		
Clusters	Years	Frequency	Clusters	Years	Frequency
One	2006	1	One	2006	1
	2007	1		2007	1
	2008	1		2008	1
	2009	1		2013	1
	2010	1		Total	4
	2011	1	Two	2009	1
	2012	1		2010	1
	2013	1		2011	1
	2014	1		2012	1
	2015	1		2014	1
	Total	10		2015	1
Two	2016	1		2016	1
	2017	1		2017	1
	2018	1		2018	1
	Total	3		Total	9

### Factor analysis

**Figure 1:**
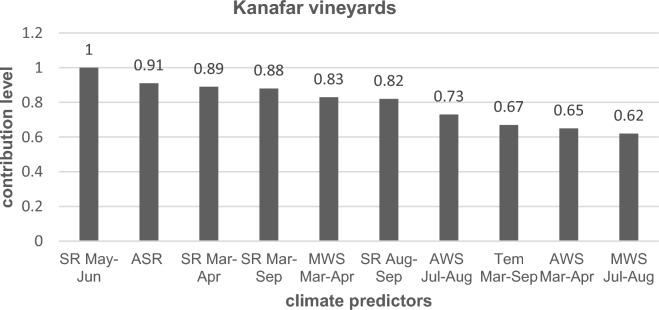
Contribution levels of significant weather predictors at Kanafar vineyards. SR: solar radiation; ASR: annual solar radiation; MWS: maximum wind speed; AWS: average wind speed, Tem: temperature.

**Figure 2:**
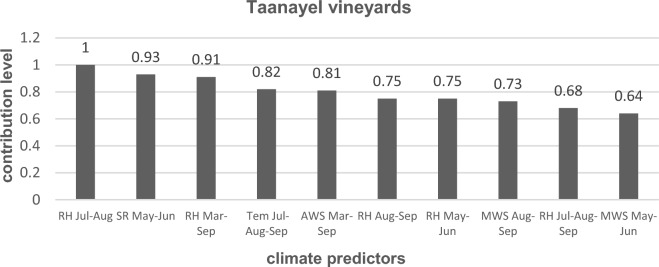
Contribution levels of significant weather predictors at Taanayel vineyards. RH: Relative humidity; SR: solar radiation; Tem: temperature; AWS: average wind speed; MWS: maximum wind speed.

Factor analysis estimated the ten most statistically significant weather predictors affecting the grouping of the 13 years of study into separate clusters at each vineyard according to the level of contribution of each predictor. In Kanafar vineyards, SR was the most influential predictor, precisely SR during late spring-early summer (SR May–Jun), followed by annual solar radiation (ASR), SR during early-mid spring (SR Mar–Apr), SR during growing season (SR Mar–Sep) in respective decreasing order of importance. Other weather variables with a lower level of contribution at Kanafar are presented in Fig. 1. In Taanayel vineyards, RH during mid-late summer (RH Jul-Aug) was the most influential weather predictor followed by SR during late spring-early summer (SR May-Jun), RH during growing season (RH Mar–Sep), temperature during mid-late summer (Tem Jul–Aug) and AWS during growing season (AWS Mar–Sep) in respective decreasing order of importance. Other contributing factors of a lower level of contribution at Taanayel are presented in Fig. 2.

At Kanafar vineyards (Table 3), the majority of weather variables differed significantly (*P*_*value*_ < 0.05) between cluster 1 and 2. Exceptions were for average temperature during the growing season (Tem Mar–Sep) and AWS during early-spring to mid-summer (AWS Mar–Apr), due to high standard deviations in these two factors, though the contribution level of those two predictors was low compared to others. Overall, SR May–Jun, ASR, SR Mar–Apr, and SR Mar–Sep recorded significantly higher values in cluster 2 (years 2016–2018) compared to cluster 1 (2006–2015), while MWS Mar–Apr, SR Aug–Sep, AWS Jul–Aug, and MWS Jul–Aug recorded significantly lower values in cluster 2.

**Table 3:** Comparison of weather variables (means ± SD) in years of cluster 1 and cluster 2 at Kanafar and Taanayel vineyards.

WV	KAN1	KAN2	*P*_*value*_	WV	TAN1	TAN2	*P*_*value*_
SRMayJun	243.1 ± 16.2	270.2 ± 3.7	0.00	RHJulAug	44.0 ± 4.4	53.8 ± 4.6	0.00
ASR	169.7 ± 10.2	183.6 ± 3.0	0.00	SRMayJun	291 ± 18.7	329.3 ± 16.8	0.00
SRMarApr	176.6 ± 7.6	190.5 ± 6.4	0.00	RHMarSep	47.6 ± 4.7	56.8 ± 5.1	0.00
SRMarSep	225.7 ± 20.9	255.9 ± 4.7	0.00	TemJulAuSe	22.2 ± 2.7	24.2 ± 2.9	0.06
MWSMarApr	3.0 ± 0.3	2.4 ± 0.2	0.00	AWSMarSep	0.8 ± 0.2	0.7 ± 0.2	0.01
SRAugSep	226 ± 21.5	225.7 ± 11	0.00	RHAugSep	51.2 ± 5.5	60.4 ± 7.7	0.00
AWSJul-Aug	0.78 ± 0.14	0.62 ± 0.12	0.00	RHMayJun	43.9 ± 5.04	52.7 ± 4.4	0.00
TemMarSep	20.2 ± 0.9	20.8 ± 1.02	0.10	MWSAugSep	6.0 ± 0.7	4.7 ± 0.6	0.00
AWSMarApr	0.76 ± 0.13	0.67 ± 0.11	0.06	RHJulAugSep	47.7 ± 4.7	57.1 ± 4.7	0.00
MWSJulAug	3.25 ± 0.35	2.87 ± 0.17	0.00	MWSMayJun	6.3 ± 0.7	5.3 ± 0.7	0.00

KAN1: Kanafar cluster 1 (2006–2015); KAN2: Kanafar cluster 2 (2016 till 2018); TAN1: Taanayel cluster 1 (2006–2008 and 2013); TAN2: Taanayel cluster 2 (2009–2018, excluding 2013); SR: solar radiation (W m^−2^); ASR: annual solar radiation; MWS: maximum wind speed (m s^−1^), tem: temperature (°C).

At Taanayel vineyards (Table 3), many of the weather variables were significantly different among both clusters of years, except for the temperature during mid-summer to early-autumn (Tem Jul–Aug–Sep). The changes in weather variables were translated by a significant increase in cluster 2 compared to cluster 1 for most of the indicators, except AWS Mar–Sep, MWS Aug–Sep, and MWS May–Jun which decreased in cluster 2.

### Changes in yield and quality by years

Results (Table 4) showed that at Kanafar vineyards, there was a reduction in average values of PA (by 1.5%), yield (by 35%), W200B (36.2 g), and TPR (by 36.2 mg GAE g^−1^ = by half) in cluster 2 years (2016–2018) compared to cluster 1. On the other hand, TA, TAP, and PAE were not significantly different at these vineyards among the two clusters of years. In Taanayel, the majority of indicators tested for Cabernet Sauvignon did not differ significantly between the two clusters of years at this vineyard, except TPR content in berries, which decreased significantly in cluster 2. High values of SD show variability of yield among years occurring at Taanayel vineyards.

**Table 4:** Comparison between Cabernet Sauvignon indicators among two clusters of years at Kanafar and Taanayel vineyards.

	KAN1	KAN2	*P*_*value*_	TAN1	TAN2	*P*_*value*_
Yield	12.985.6 ± 4143	8439.8 ± 1604.0	0.00	8723.3 ± 1906.3	9109.6 ± 4160.6	0.7
W200B	236.5 ± 33.7	202.3 ± 9.4	0.00	777.4 ± 210.5	831.5 ± 216.0	0.5
PA	14.3 ± 1.1	12.8 ± 0.7	0.00	14.0 ± 0.7	13.7 ± 0.7	0.19
TA	3.8 ± 0.6	4.3 ± 0.6	0.14	4.1 ± 0.7	4.1 ± 0.6	0.9
TAP	934.9 ± 305.8	963.1 ± 90.5	0.08	239.4 ± 23.9	227.8 ± 34.0	0.3
PAE	57.7 ± 11.9	59.6 ± 16.9	0.07	58.4 ± 12.9	58.4 ± 14.9	0.9
TPR	72.8 ± 19.3	36.6 ± 6.1	0.00	75.9 ± 19.9	54.2 ± 29.8	0.03

KAN1: Kanafar cluster 1 (2006–2015); KAN2: Kanafar cluster 2 (2016 till 2018); TAN1: Taanayel cluster 1 (2006–2008 and 2013); TAN2: Taanayel cluster 2 (2009–2018, excluding 2013); Yield in kg ha^−1^, W200B: average weight of 200 berries (g); PA: potential alcohol (%); TA: titratable acidity (g L^−1^); TAP: total anthocyanin potential (mg kg^−1^); PAE: potential anthocyanin extractibility (%); TPR: total polyphenolic richness (mg GAE g^−1^).

Grapes’ anthocyanins are responsible for the colour of the wine produced from Cabernet Sauvignon. Their accumulation in grape skins is known to be affected by climate conditions at the vineyards, such as light and temperature^21,22^, and also by additional factors like soil type and the nutrient supply to vines^23,24^, cultural practices, and training systems^25^. Therefore, at both studied vineyards, anthocyanins’ accumulation could be more affected by the latter factors rather than by climate conditions.

Pairwise comparison of means (Table 5) showed that at Kanafar vineyards, average yield peaked in 2009 (19,187.0 kg ha^−1^), and recorded the lowest value in 2015 (6235.0 kg ha^−1^), whereas in Taanayel, average yield was the highest in 2011 (14,279.0 kg ha^−1^) and the lowest in 2017 (2627.0 kg ha^−1^). The data show that the years 2009 and 2011 marked a turning point, after which yield was significantly reduced at Kanafar and Taanayel vineyards, respectively (by 31–67% in Kanafar and 14–82% in Taanayel). Further, Cabernet Sauvignon yields were significantly lower in Taanayel compared to Kanafar in most years, except in 2010, 2011, and 2014. Berry weights (AW200B) peaked in 2010 (293.7 g) at Kanafar, and in 2015 in Taanayel (267.4 g) and were significantly reduced in the following years at both vineyards. This indicator did not differ significantly between both vineyards in the majority of years, with minor exceptions.

**Table 5:** Effect of years on productive and qualitative indicators of Cabernet Sauvignon at Kanafar and Taanayel vineyards.

	Yield	W200B	PA	TA	TAP	PAE	TPR
KAN2006	18,494.0s	261.8hijk	14.3abcd	4.0ab	760.53cde	41.4bc	92.73hi
KAN2007	16,204.8r	269.2kl	13.1ab	3.5a	659.7bc	67.8ghij	75.7fg
KAN2008	15,012.0q	247.8fghijk	13.3abcd	3.9ab	891.4fgh	69.8hij	88.7hi
KAN2009	19,187.0s	234.9defghij	14.5bcd	4.1ab	675.2bc	60.5efghi	74.3fg
KAN2010	9602.0jk	293.7l	14.3 bcd	4.6ab	1102.8jk	70.1hij	73.23fg
KAN2011	12,783.0op	234.4defghi	16.2e	3.8ab	599.8b	56.3defg	58.03e
KAN2012	13,235.0p	225.6cdefg	14.8d	3.4a	885.8fgh	52.6cdef	84.03gh
KAN2013	9958.0jkl	204.0bcd	14.7cd	3.8ab	1644.4l	37.5ab	95.8i
KAN2014	9145.0ij	181.8ab	14.3abcd	4.0ab	1185.8k	61.8fghi	48.17bcde
KAN2015	6235.0c	211.7bcde	13.2abc	3.3a	944.3ghi	59.6efghi	38.03abc
KAN2016	10,434.0kl	202.8abcd	12.8a	4.2ab	978.3hi	54.1def	37.13ab
KAN2017	6825.0cd	198.4abc	12.8a	4.1ab	874.5fgh	80.1j	42.97bcd
KAN2018	8060.3efg	205.7bcd	12.8a	4.4ab	1036.4ij	44.7bcd	29.87a
TAA2006	7619.5cde	255.6ghijk	14.2abcd	3.9ab	901.6fgh	68.4ghij	96.43i
TAA2007	7188.8cd	266.5ijkl	13.3abcd	3.9ab	580.8b	72.2ij	88.37hi
TAA2008	11,566.0mn	216.9cdef	14.1abcd	3.9ab	589.9b	44.5bcd	49.73de
TAA2009	12,634.0 op	253ghijk	13.8abcd	4.5ab	680.8bc	26.9a	89.03hi
TAA2010	10,858.0lm	172.9a	13.8abcd	4.3ab	1097.3jk	71.6hij	112.0f
TAAl2011	14,279.0q	242.7efghijk	13.6abcd	3.9ab	816.2def	62.0fghi	72.97fg
TAA2012	12,226.0no	244.5efghijk	13.9abcd	4.6ab	452.5a	63.1fghi	46.97bcde
TAA2013	8519.0ghi	218.7cdef	14.6cd	4.8b	1037.4ij	48.4bcde	69.12f
TAA2014	11,830.9no	206.2bcd	13.9abcd	4ab	1176.0k	63.3fghi	49.03cde
TAA2015	4099.0b	267.4jkl	13.7abcd	3.5a	830.8efg	45.3 bcd	29.10a
TAA2016	8990.0hij	230.3cdefgh	13.3abcd	3.9ab	829.6efg	59.3efgh	30.6a
TAA2017	2627.0a	219.6cdef	14.3bcd	4.1ab	716.0cd	71.1hij	28.03a
TAA2018	4442.7b	213.5bcde	13.0ab	4.6ab	884.5fgh	62.4fghi	30.0a

KAN: Kanafar vineyards; TAN: Taanayel vineyards, Yield in kg ha^−1^; W200B: average weight of 200 berries (g); PA: potential alcohol percentage (%); TA: titratable acidity (g L^−1^); TAP: total anthocyanin potential (mg kg^−1^)’; PAE: potential anthocyanins extractibility (%); TPR: total polyphenolic richness (mg GAE g^−1^).

Potential alcohol (PA) in berries did not differ significantly between years at both vineyards, however in Kanafar, a significant increase in this indicator occurred in 2011 recording 16.2%. Overall, TAP values recorded in Taanayel were comparable or lower to that recorded in Kanafar, and were significantly higher in the years 2010, 2013, and 2014 compared to other years at both vineyards. Total Polyphenolic Richness (TPR) ranges were 29.9–95.8 mg/kg in Kanafar, and 28.0–112.0 mg/kg in Taanayel. Though TPR values were significantly different in the majority of years at both vineyards, they followed a more or less progressive decreasing pattern with consecutive years at both vineyards with minor exceptions (2013 in Kanafar, and 2010 in Taanayel).

### Effect of weather conditions

Since variations in average yield, W200B, PA, and TPR between cluster 1 and 2 of years were detected at Kanafar, and variation of TPR at Taanayel, a multi-linear regression analysis was performed to correlate these variations to those of the most influential weather variables at each vineyard. Results of the multi-linear regression analysis (Table 6) suggest that the model built for the indicator yield is able to describe 60% (*R*^2^ = 0.59) of the variations in Cabernet Sauvignon yields. The sign of the coefficients indicates the direction of change in the yield versus changes in weather variables. Yield at Kanafar was negatively correlated with SR Mar–Apr, SR May–Jun, Tem Mar–Sep, and MWS Jul–Aug. As yield was lower in cluster 2 years, such a decrease may be caused by higher temperature during the growing season (by 0.6 °C for Tem Mar–Sep) and higher solar radiation from early spring to early summer (by 13.9 W m^−2^ for SR Mar–Apr and 27.1 W m^−2^ for (SR May–Jun), and to lower values of MWS during mid-late summer (by 0.4 m s^−1^ for MWS Jul–Aug), which occurred during the years 2016, 2017, and 2018 at Kanafar. Pagay and Collins^26^ reported earlier negative effects of an increase in the average growing season temperature on grape yield and quality.

**Table 6:** Multivariate regression analysis of Cabernet Sauvignon indicators in relation to weather variables.

Kanafar											
	ASR	SRMarApr	SRMayJun	SRAugSep	SRMarSep	AWSMarApr	MWSMarApr	AWSJulAug	T MarSep	MWSJulAug	***R***^**2**^
Yield											
* Cf*	1.05	− 0.46	− 1.60	0.39	0.54	− 1.81	0.44	0.09	− 1.05	− 0.12	0.59
* p*	0.00	0.25	0.00	0.37	0.15	0.30	0.09	0.67	0.64	0.63	
W200B											
* Cf*	2.07	− 0.67	− 0.67	0.46	− 0.26	− 0.05	0.32	0.10	0.32	− 0.34	0.34
* p*	0.05	0.18	0.32	0.40	0.58	0.81	0.32	0.7	0.27	0.29	
PA											
* Cf*	0.89	− 0.41	− 0.41	0.79	− 0.01	− 0.39	0.95	0.34	− 0.54	0.14	0.61
* p*	0.01	0.29	0.42	0.07	0.98	0.03	0.00	0.09	0.02	0.56	
TPR											
* Cf*	1.21	− 0.27	− 1.29	0.09	0.81	− 0.25	0.84	− 0.09	− 0.08	0.28	0.58
* p*	0.00	0.58	0.02	0.82	0.04	0.16	0.00	0.64	0.73	0.27	

Taanayel											
	RHJulAug	SRMayJun	RHMarSep	TJulAugSep	AWSMarSep	RHAugSep	RHMayJun	MWSAugSep	RH JulAugSep	MWSMayJun	*R*^2^
TPR											
* Cf*	− 0.22	0.06	− 0.01	− 0.27	− 0.15	0.27	0.17	0.63	− 0.02	0.05	0.3
* p*	0.68	0.85	0.97	0.37	0.5	0.48	0.68	0.09	0.9	0.9	

*Cf. Regression coefficient; p: p-value;* ASR: annual solar radiation; AWS; average wind speed; MWS: maximum wind speed; T: temperature; RH: relative humidity; W200B: average weight of 200 berries; PA: potential alcohol; TA: titratable acidity; PAE: potential anthocyanin extractibility; TAP: total anthocyanins potential; TPR: total polyphenolic richness.

In the case of W200B recorded in Kanafar and TPR recorded in Taanayel, weather variables account only for 34% (*R*^2^ = 0.34) of changes in this indicator, while 66% of these variations are explained by other factors, which are most likely due to cultural practices adopted at the vineyard, like irrigation, hormones spraying^27^, fruit thinning^28^, timing and method of leaf removal^29^, etc.

Furthermore, weather variables account for 61% (*R*^2^ = 0.61) of the variation in PA and for 58% (*R*^2^ = 0.58) of the variation in TPR of berries at Kanafar. Considering the signs of correlations, PA was positively correlated with wind speed during mid-late summer (AWS Jul–Aug and MWS Jul–Aug), and negatively correlated with solar radiation intensity and temperature during the growing season (SR Mar–Sep and Tem Mar–Sep), and with SR from early spring to early-summer (SRMarApr and SRMayJun). TPR in berries followed similar relationships with weather variables as PA, except for a positive correlation with solar radiation during the growing season (SR Mar–Sep).

Earlier work of Navrátilová et al.^30^ reported a significant positive correlation between the gradual increase of average temperatures in the growing season and the increase in sugar accumulation in berries, contradicting our findings. Also, Schultz^31^ speculated that long-term temperature increases have had a demonstrable effect on grape composition, as the sugar concentration increases and acidity decreases. Sugar accumulation is also influenced by the timing of veraison, which is affected by both cultivar genetics and environmental conditions prior to veraison^32^. In warmer climates, ripening will occur earlier and will possibly affect vintage quality by a quick increase in fruit maturity^14^. Extreme temperatures during ripening can reduce quality due to the excessive sugar levels and low acidity, along with changes in anthocyanin and flavonoid concentrations^33,34^. Biosynthesis, translocation, degradation and accumulation of substances in the berry are transferred to the wine, defining its color, aroma, and flavor^35^.

Light is also a critical factor which affects sugar accumulation and berry ripening, and consequently impacting grape quality^36^. However, too high light intensity would damage the berry, thus influencing the quality of grapes^37^. Also, the level of sugars and aromatic compounds in grapes depend on microclimatic conditions around the cluster zone^38–40^. Overall, SR and temperature increased during the growing season at Kanafar, but wind activity decreased in cluster 2 during mid-late summer, however sugar accumulation was reduced in berries compared to cluster 1. Such finding contradicts much of the discussion above. Also, though TPR in berries was positively correlated with solar radiation during the growing season and during early-spring to late-summer, it decreased in cluster 2 at Kanafar. According to Martínez-Lüscher et al.^41^ when high air temperature and excessive radiation combine, detrimental effects on flavonoid content may occur in warm climate regions. Polyphenols are responsible for wine color and stability, for wine longevity thanks to their antioxidant activity, and for wine’s oral characteristics^42^. Therefore, changing climate conditions at both vineyards will likely cause negative consequences on the quality of wine produced at Kanafar and Taanayel. Earlier, Muñoz et al.^43^ reported varying levels of anthocyanins in berries when Cabernet Sauvignon vines were cultivated in vineyards with different geographical indications of Mendoza; anthocyanin accumulation in berries increased at high altitudes compared to low altitude environments due to increased UV-B exposure. Moreover, University of California, Davis researchers; Martínez-Lüscher et al.^44^ suggested that the single high-wire trellis systems is an effective method in mitigating the impact of heat waves and exposure of berries in Cabernet sauvignon. According to them, the solution to protect fragile grapes from increasingly hot environments lies within the grapevine trellis system.

## Conclusions

Cabernet Sauvignon yields dramatically decreased after 2009 in Kanafar and after 2011 in Taanayel. Changing weather conditions had much greater negative influences on yield and yield components in Kanafar than in Taanayel, but had a similar negative influence on polyphenol accumulations, thus on berry quality at both vineyards. Various adaptation measures may be taken to cope with changing weather conditions at these vineyards, such as irrigation management to maintain proper soil moisture and promote transpiration, responsible canopy management around the fruit zone to improve air circulation and allow adequate exposure to sunlight, evaporative cooling to cool the canopy and fruit, and adjusting planting density and trellising systems to regulate fruit temperature during hot periods.

## Data Availability

The datasets generated and/or analysed during the current study are not publicly available due the confidentiality reasons, but are available from the corresponding author on reasonable request.
